# The diaphragm has an expiratory braking effect in spontaneously breathing lung injured animals as shown by electrical diaphragmatic activity

**DOI:** 10.1186/2197-425X-3-S1-A572

**Published:** 2015-10-01

**Authors:** M Pellegrini, G Hedenstierna, A Roneus, M Segelsjö, A Larsson, G Perchiazzi

**Affiliations:** Department of Surgical Sciences, Hedenstierna Laboratory - Uppsala University, Uppsala, Sweden; Department of Emergency and Organ Transplant, Bari University, Bari, Italy; Department of Medical Sciences, Hedenstierna Laboratory - Uppsala University, Uppsala, Sweden; Department of Surgical Sciences, Section of Radiology - Uppsala University, Uppsala, Sweden

## Introduction

Albeit defined as an inspiratory muscle, the diaphragm interacts with the respiratory system also during expiration. The expiratory load on the diaphragm derives from lung volume and hence, possibly from lung collapse. We hypothesized that the diaphragm has a braking effect during the whole expiration, preserving lung patency during SB in collapse prone lungs. We investigated this hypothesis by measuring the expiratory electrical diaphragmatic activity (EAdi_exp_) in the modulation of expiratory flow (V'exp) in a porcine model.

## Methods

Mild acute respiratory distress syndrome (ARDS) was induced in 7 anesthetized, tracheostomized pigs by repeated lung lavages, targeting a PaO_2_/FiO_2_ of 250 mmHg. After stabilization, the animals were converted to spontaneous breathing (SB) and underwent a decremental continuous positive airway pressure (CPAP) trial of 15, 12, 9, 6, 3 and 0 cmH_2_O, while EAdi_exp_ and the expiratory trans-diaphragmatic pressure (Pdi_exp_) were measured. In two of the pigs EAdi_exp_ and Pdi_exp_ were assessed also during controlled mechanical ventilation (CMV) after muscle relaxation using the same positive end-expiratory pressure (PEEP) as for CPAP. For each studied condition, lung volume was assessed by profiting of sulfur hexafluoride (SF_6_) washin/washout technique. The V'exp slope before the expiratory peak flow and the expiratory volume (Vol_exp_) at 50% and 75% of expiratory time were estimated. Statistical significance of expiratory EAdi_exp_/Pdi_exp_ was assessed by F-tests (α = 0.05). Between SB and CMV, comparisons were performed at the same lung volume, which was verified by applying the Kolmogorov-Smirnov test (α=0.05).

## Results

When CPAP was decreased, EAdi_exp_ increased until a CPAP of 6 cmH_2_O and then remained unaltered (although elevated) with further decrease in CPAP. EAdi_exp_ and Pdi_exp_ were tightly correlated as confirmed by an R^2^ > 0.82 (p < 0.01). Lung volumes were comparable during SB and CMV when exposed to the same CPAP/PEEP levels. V'exp had a slower initial increase (before reaching its peak) during SB than during CMV. By decreasing CPAP/PEEP, V'exp rise was markedly more delayed during SB than during CMV at the same lung volumes indicating a delayed emptying of the lungs, which was also shown by that Vol_exp_ at 50% and at 75% of expiration were higher during SB than during CMV.

## Conclusions

The findings suggest that the diaphragm plays an important role during expiration. The EAdi_exp_ modulates diaphragmatic expiratory mechanical “braking” activity, possibly to protect against reductions in lung volume and atelectasis formation. The EAdi_exp_ can be useful in setting CPAP/PEEP during SB in mild ARDS conditions.

## Grant Acknowledgment

The School of Anesthesia and Intensive Care of Medicine, Bari University, Italy; The Swedish Heart and Lung Foundation.

